# Sinal do chifre de búfalo – Um novo achado em RM para rupturas em alça de balde do menisco

**DOI:** 10.1055/s-0045-1809514

**Published:** 2025-06-23

**Authors:** Rafael R. Pereira, João Cabral, João Janeiro, José Padín, Joaquim Soares do Brito, Rodrigo A. Goes

**Affiliations:** 1 Serviço de Ortopedia, Unidade Local de Saúde Santa Maria, Lisboa, Portugal; 2 Unidade de Cirurgia de Joelho e Tornozelo, Centro de Ortopedia e Traumatologia, Hospital CUF Descobertas, Lisboa, Portugal; 3 Serviço de Radiologia, Unidade Local de Saúde Santa Maria, Lisboa, Portugal; 4 Faculdade de Medicina da Universidade de Lisboa, Lisboa, Portugal

**Keywords:** diagnóstico por imagem, imagem por ressonância magnética, joelho, lesões do menisco tibial, diagnostic imaging, knee, magnetic resonance imaging, tibial meniscus injuries

## Abstract

**Objetivo:**

Descrever um novo sinal em imagens axiais de ressonância magnética (RM) de pacientes com rupturas em alça de balde do menisco.

**Métodos:**

De 610 pacientes consecutivos com diagnóstico cirúrgico de ruptura do menisco, aqueles com padrão em alça de balde foram escolhidos, e 28 atenderam aos critérios de inclusão. O mecanismo de lesão mais frequente foi a torção com ou sem estresse coronal (16 pacientes). Além disso, a lesão foi relacionada ao esporte em 12 casos. Todos os pacientes eram sintomáticos e tinham radiografias que mostravam a preservação da linha articular. Em seguida, seus exames de RM foram analisados.

**Resultados:**

O padrão de chifre de búfalo foi encontrado em 13 pacientes (46,4%) no menisco medial ou lateral. Foi o 3 ^o^ sinal mais prevalente, depois do fragmento no interior da incisura intercondilar ( *n* = 21; 75,0%) e da ausência do sinal da gravata borboleta ( *n* = 17; 60,7%). Observamos uma associação significativa a outros sinais de deslocamento da alça do menisco. O sinal não foi encontrado em meniscos saudáveis, nem foi afetado pela ocorrência de ruptura do ligamento cruzado anterior.

**Conclusão:**

O chifre de búfalo é um novo achado para rupturas em alça de balde do menisco com deslocamento; é fácil de identificar e relevante na interpretação de imagens de RM de corte axial. Seu reconhecimento é muito importante para determinar o tipo de tratamento e o plano cirúrgico.

## Abstract (en)

**Objective:**

To describe a new sign on magnetic resonance imaging (MRI) axial images of patients with bucket-handle meniscal tears.

**Methods:**

Of 610 consecutive patients with a surgical diagnosis of meniscal tear, those with a bucket-handle pattern were chosen, and 28 met the inclusion criteria. The most frequent mechanism was a twisting injury with or without a coronal stress (16 patients), and the injury was sports-related in 12 cases. All patients were symptomatic and had X-rays showing a preserved joint line. Next, their MRI examinations were analyzed.

**Results:**

The buffalo horn pattern was found in 13 patients (46.4%), occurring in either the medial or the lateral meniscus. It was the 3 ^rd^ most prevalent sign, after the fragment within the intercondylar notch ( *n* = 21; 75.0%) and the absent bow tie sign ( *n* = 17; 60.7%). We observed that it had a significant association with other signs of displaced meniscal handle. The sign was neither found on the healthy menisci, nor was affected by the occurrence of an anterior cruciate ligament tear.

**Conclusion:**

The buffalo horn is a new finding for displaced meniscal bucket-handle tears; it is easy to identify and relevant in the interpretation of axial MRI images. Its recognition is very important to determine the type of treatment and the surgical plan.

## Introdução

Os meniscos são lâminas fibrocartilaginosas intracapsulares em forma de crescente que atuam na transmissão de carga, absorção de choque, estabilidade, lubrificação, difusão de nutrientes, percepção sensorial e propriocepção. 1 As rupturas de menisco são comuns, com incidência relatada de cerca de 60 casos por 100 mil habitantes nos Estados Unidos. 2 Há diversos padrões. As rupturas em alça de balde consistem em uma ruptura longitudinal de espessura total que se propaga anterior e posteriormente, criando um fragmento interno – a “alça” – que pode se deslocar para o interior da incisura intercondilar. 3 Estas lesões representam aproximadamente 10% de todas as rupturas, 3 4 e ocorrem principalmente no menisco medial, 4 5 mas podem afetar o menisco lateral. 6 7 8 Como o tratamento cirúrgico é frequentemente necessário, o diagnóstico pré-operatório correto é importante para otimizar o tratamento e salvar o tecido do menisco. 9 A ressonância magnética (RM) é o método padrão-ouro de diagnóstico por imagem, com uma sensibilidade geral de até 90,0% e especificidade de até 89,0%. 10 11 12 13 Na RM, as rupturas em alça de balde do menisco tendem a apresentar alguns sinais bem conhecidos, principalmente em cortes coronais e sagitais, como a ausência do sinal da gravata borboleta, 14 o sinal do ligamento cruzado posterior (LCP) duplo, 15 16 o sinal do corno anterior duplo, 17 do sinal do menisco invertido, 18 o sinal do corno posterior desproporcional, 19 o sinal do ligamento cruzado anterior (LCA) duplo, 20 o sinal do LCP triplo, 7 o sinal cruzado triplo, 8 o sinal cruzado quádruplo, 6 e a presença de um fragmento no interior da incisura intercondilar 4 ( Anexo A ). A sensibilidade e a especificidade relatadas para o diagnóstico de rupturas em alça de balde do menisco são bastante variáveis, de 64,0% a 93,0% 4 5 21 e de 64,0% a 100%, 10 22 respectivamente, mas melhoram se mais sinais forem conhecidos. 5 22

**Anexo A:** Definição dos achados característicos à ressonância magnética de rupturas em alça de balde do menisco

Autores	Sinal	Definição
Weiss et al., 1991; 15 Singson et al., 1991 16	LCP duplo	Uma banda de baixo sinal anterior e paralela ao ligamento cruzado posterior em imagens sagitais.
Haramati et al., 1993 18	Menisco invertido	Um corno meniscal anterior com aumento anormal (> 6 mm).
Wright et al., 1995 4	Fragmento intercondilar	Uma área semelhante a uma faixa de baixa intensidade de sinal no interior da incisura, mas que não aparece no mesmo corte do que o LCP.
Helms et al., 1998 14	Sinal da ausência de gravata borboleta	A ocorrência de apenas um ou nenhum segmento do corpo meniscal em imagens sagitais consecutivas de ressonância magnética.
Ruff et al., 1998 17	Corno anterior duplo	Presença de dois triângulos não justapostos verticalmente, mas localizados lado a lado no mesmo plano horizontal em um corte sagital, parecendo dois cornos anteriores do menisco.
Chen et al., 2001 19	Corno posterior desproporcional	Corno posterior no corte central maior do que no corte periférico nas imagens de ressonância magnética sagital.
Bugnone et al., 2005 6	Sinal cruzado quádruplo	Quatro estruturas na incisura intercondilar observadas em cortes coronais consecutivos: ambos os fragmentos deslocados de meniscos rompidos, o coto do LCA rompido e o LCP intacto.
Bui-Mansfield et al., 2006 20	LCA duplo	A presença do fragmento imediatamente posterior ao LCA.
Kakel et al., 2010 7	LCP triplo	Presença de um LCP intacto e dois fragmentos deslocados na incisura intercondilar devido a duas rupturas em alça de balde em um corte sagital de um joelho com deficiência do LCA.
Rao et al., 2012 23	Sinal em V	O “V” é visto na junção do fragmento deslocado (alça), pois forma um ângulo reto com o menisco, que está no local correto.
Sales et al., 2021 8	Sinal cruzado triplo	Três estruturas na incisura intercondilar observadas em cortes coronais: fragmentos deslocados de meniscos rompidos e o LCP intacto.
Barrie, 1979 25	Cisto parameniscal	Acúmulo de fluido em íntima relação com o menisco, seja por contato direto ou fístula.
Gale et al., 1999 26	Extrusão de menisco	Quantificada em imagem coronal em seu maior valor e considerada quando a margem periférica do menisco se estende 3 mm ou mais além da borda do platô tibial.
Kaplan et al., 1999 27	Edema da medula subcondral	Edema não linear sem margem claramente definida.
Kolman et al., 2004 24	Efusão articular	Uma medida anteroposterior de 10 mm ou mais na bolsa suprapatelar lateral, considerada anormal.
Bergin et al., 2008 28	Edema linear da medula subcondral	Edema bem delimitado, paralelo à superfície articular e com menos de 5 mm de profundidade.

**Abreviaturas:** LCA, ligamento cruzado anterior; LCP, ligamento cruzado posterior.

Pelo que sabemos, havia apenas um sinal descrito na RM axial: o sinal em V. 23 Este artigo teve como objetivo relatar um novo achado a ser identificado nessa projeção, com aparência de chifre de búfalo e observado em pacientes com rupturas em alça de balde do menisco. Além disso, este estudo determinou a sensibilidade desse achado e a comparou à de outros sinais já conhecidos.

## Materiais e Métodos

### Conformidade com os Padrões Éticos

Este estudo obedece aos padrões éticos do Comitê de Pesquisa institucional e da Declaração de Helsinque de 1964 e suas emendas posteriores ou padrão ético comparável. O estudo recebeu a aprovação n. 215/22 do Conselho de Ética do Centro Acadêmico Médico de Lisboa. Não havia necessidade de obtenção de consentimento livre e esclarecido desde que os padrões de proteção de dados pessoais fossem atendidos, mas obtivemos o consentimento por escrito de todos os pacientes cujas imagens de RM foram incluídas neste artigo.

Este é um estudo retrospectivo baseado em exames de RM de pacientes diagnosticados durante a cirurgia com rupturas em alça de balde do menisco, desconsiderando idade, sexo, mecanismo de trauma, tempo entre o trauma e a RM e entre o trauma e a cirurgia, tratamento, cirurgião, lesão do LCA, e joelho ou menisco acometido.

**Fig. 1:**
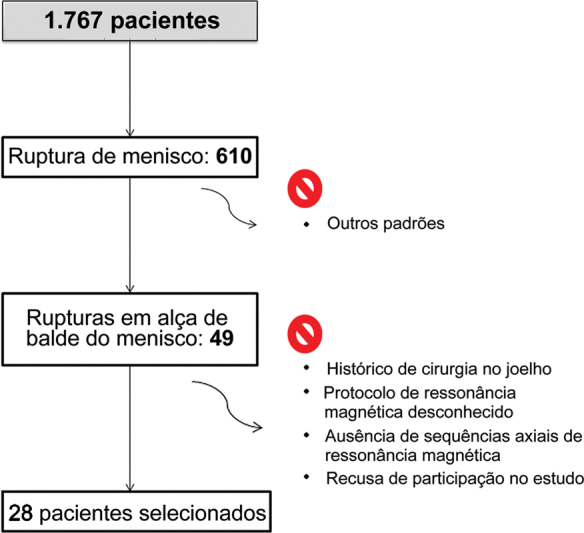
Fluxograma que mostra a estratégia de busca e os critérios de seleção.

Obtivemos uma amostra consecutiva de 1.767 pacientes operados no Serviço de Ortopedia de um hospital universitário por qualquer patologia do joelho entre 2012 e 2021 ( Fig. 1 ). Este período foi escolhido por conter o maior número de pacientes disponíveis em nossa instituição devido à limitada informatização dos processos médicos nos anos anteriores. Apenas 610 pacientes apresentaram ruptura de menisco comprovada cirurgicamente, e 49 tinham padrão em alça de balde. Em seguida, foram excluídos os pacientes com os seguintes critérios: histórico de cirurgia no joelho, outros padrões de ruptura de menisco, ausência de ruptura de menisco, protocolo de RM desconhecido, sequências axiais ausentes ou sem aquisição de meniscos, e recusa em participar desta pesquisa. A amostra coletada incluiu 28 pacientes, 22 homens e 6 mulheres, com média de idade de 34,2 (± 14,0; intervalo: 9–63) anos. O mecanismo de lesão mais frequente relatado pelos pacientes foi a torção com ou sem carga coronal (16 pacientes). Quatro pacientes apresentaram a lesão após flexão do joelho ou agachamento. Dois casos sofreram queda da própria altura, e um descreveu um trauma complexo durante a prática de surfe. Cinco pacientes não conseguiram identificar nenhum trauma.

Doze destas lesões foram relacionadas a esportes. Todos os pacientes apresentaram, em algum grau, dor no joelho, derrame articular, sensação de travamento ou laxidez, ou perda de extensão. Todas as radiografias demonstraram a preservação da linha articular.

Ao todo, 24 exames de RM foram realizados no hospital em que este estudo foi conduzido e 4 foram realizados em outro lugar, mas com protocolo semelhante. Dezessete utilizaram o *scanner* Philips Intera de 1,5 T, e 11, o *scanner* Philips Achieva de 3,0 T. A bobina de joelho específica para recepção fornecida pelo fabricante para cada *scanner* foi utilizada. As aquisições padrão estão resumidas na Tabela 1 , e incluem: coronal T1, T2 *fast field echo* (FFE) e *short tau inversion recovery* (STIR); sagital com densidade de prótons (DP) com e sem supressão de gordura; e axial T2 FFE. Alguns exames incluíram imagens coronais T2 *spectral attenuated inversion recovery* (SPAIR) e axiais SPAIR. Um protocolo ligeiramente diferente do *scanner* Intera foi usado para avaliar o paciente pediátrico, com cortes de 3 mm e *gap* de 0,3 mm.

**Tabela 1:** Protocolos de aquisição de ressonância magnética

*Philips Intera (1,5 T)*							
	Matriz	TR/TE (ms)	Corte (mm)	*Gap* (mm)	Média	FOV (mm)	FA (°)
Coronal							
T1	400 × 300	500/22	3,5	0,35	2	180	90
T2 FFE	300 × 250	500/14	3,5	0,35	2	180	25
STIR	250 × 200	5.000/80	3,5	0,35	3	180	–
T2 SPAIR	300 × 250	3.000/60	3,5	0,35	2	180	90
Sagital							
DP	300 × 250	2.500/8	3,5	0,35	2	180	90
DP SPAIR	250 × 250	3.000/30	3,5	0,35	3	180	90
T2	300 × 250	2.500/120	3,5	0,35	2	180	90
Axial							
T2 FFE	250 × 200	600/14	3,5	0,35	2	180	25
SPAIR	250 × 200	3.000/30	3,5	0,35	4	180	90
*Phillips Achieva (3 T)*							
Coronal							
T1	400 × 350	600/20	3	0,3	2	180	90
T2 FFE	400 × 300	450/12	3	0,3	2	180	20
STIR	250 × 200	4.000/80	3,5	0,35	2	180	–
T2 SPAIR	300 × 250	4.000/65	3	0,3	2	180	90
Sagital							
DP	400 × 300	7.000/9	3	0,3	1	180	90
DP SPAIR	300 × 300	5.000/30	3	0,3	2	180	90
T2	400 × 300	7.000/140	3	0,3	1	180	90
Axial							
T2 FFE	300 × 250	500/12	3	0,3	2	180	20
SPAIR	300 × 250	3.800/65	3	0,3	2	180	90

**Abreviaturas:** FA, *flip angle* (ângulo de inclinação); FFE, *fast field echo* ; FOV, *field of view* (campo de visão); DP, densidade de prótons; SPAIR, *spectral attenuated inversion recovery* ; STIR, *short tau inversion recovery* ; TE, tempo de eco; TR, tempo de repetição.

Todas as cirurgias foram realizadas por cirurgiões de joelho experientes, com especialização. A ruptura do menisco em alça de balde foi definida como “uma ruptura longitudinal com migração central do fragmento ‘interno’ da alça”. 3

**Fig. 2:**
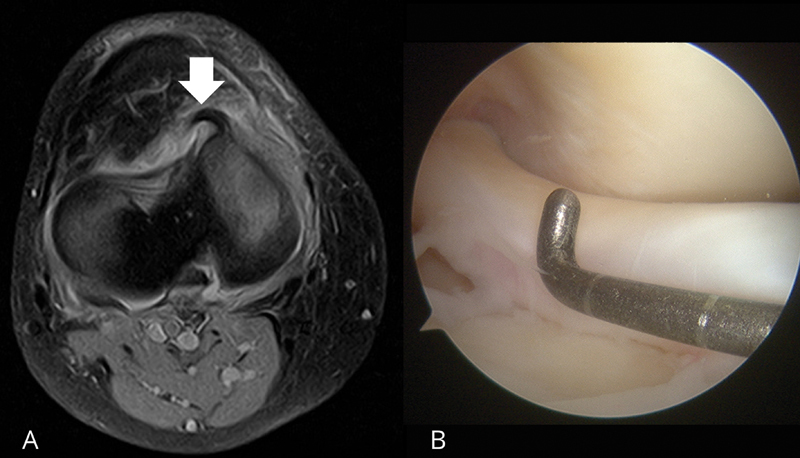
( **A** ) Imagem de ressonância magnética pré-operatória de um homem de 54 anos com ruptura em alça de balde do menisco medial do joelho direito relatada à cirurgia, que mostra o sinal do chifre de búfalo (seta) no corte transversal axial. Este achado é uma área de baixa intensidade de sinal, que se projeta da borda anterior do platô tibial medial e se assemelha a um chifre. ( **B** ) Artroscopia do joelho direito usando um portal anterolateral e mostrando o fragmento deslocado do menisco do mesmo paciente, anterior ao côndilo femoral. Sequência *spectral attenuated inversion recovery* (SPAIR) axial: matriz, 250 × 200; tempo de repetição/tempo de eco (TR/TE), 3.000/30 milissegundos; corte, 3,5 mm; *gap* , 0,35; média, 4; campo de visão ( *field of view* , FOV, em inglês), 180 mm; ângulo de inclinação ( *flip angle* , FA, em inglês), 90°.

**Fig. 3:**
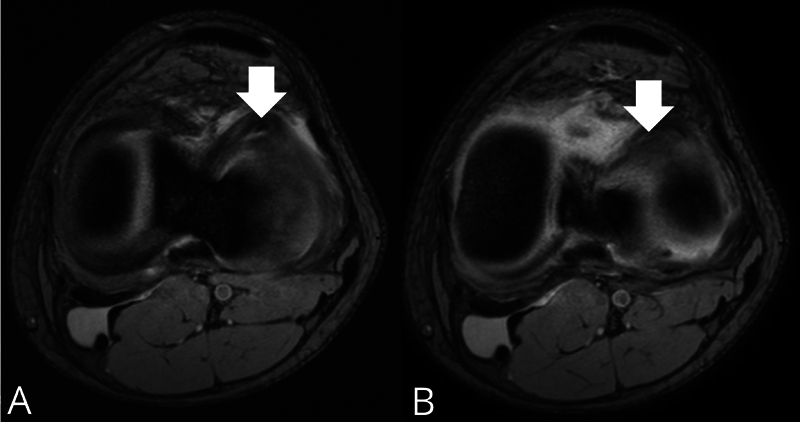
Imagem de ressonância magnética pré-operatória de um homem de 17 anos com ruptura em alça de balde do menisco lateral do joelho esquerdo relatada à cirurgia. ( **A,B** ) Corte transversal axial em T2 *fast field echo* (FFE), que mostra o sinal do chifre de búfalo (seta) no menisco lateral. O sinal tem formato de chifre, baixa intensidade, e é paralelo à borda anterior do platô tibial lateral em duas imagens axiais consecutivas. Sequência T2 FFE axial, matriz, 300 × 250; TR/TE, 500/12 milissegundos; corte, 3 mm; FOV, 180 mm, FA, 20°.

Um cirurgião sênior de joelho e um residente ortopédico foram instruídos sobre a interpretação de RM de rupturas em alça de balde, e, então, de forma prospectiva e cega, avaliaram as imagens. O consenso foi dado pelo radiologista musculoesquelético sênior. Cada exame de RM foi avaliado quanto à presença do sinal de gravata borboleta 14 ausente do sinal de LCP duplo, 15 16 do sinal do corno anterior duplo, 17 do sinal do menisco invertido, 18 do sinal do corno posterior desproporcional, 19 do sinal do LCA duplo, 20 do sinal do LCP triplo, 7 do sinal cruzado triplo, 8 do sinal cruzado quádruplo, 6 e de um fragmento no interior da incisura intercondilar. 4 Em corte transversal, o sinal em V 23 e o novo sinal foram registrados. O sinal do chifre de búfalo é a presença de uma área de baixa intensidade que se projeta da borda anterior do platô tibial medial, à semelhança de um chifre, que pode ser visto na RM axial, como demonstrado na Fig. 2 . Em caso de acometimento do menisco lateral, o sinal aparece como uma faixa em forma de chifre de baixa intensidade, paralela à borda anterior do platô tibial lateral em duas imagens axiais consecutivas ( Fig. 3 ). As outras definições usadas são revistas no Anexo A . A presença de efusão articular, 24 cisto parameniscal, 25 extrusão de menisco 26 e edema de medula óssea e sua localização 27 28 também foram registradas. À RM, os casos de suspeita de ruptura em hemialça de balde do menisco foram contados. 29 Evidências cirúrgicas de ruptura do LCA foram relatadas.

Uma pesquisa bibliográfica pelos termos e expressões *meniscos* , *alça de balde* e *imagem de ressonância magnética* foi conduzida no motor de busca PubMed, considerando apenas textos em inglês e estudos em humanos. Nenhum relato sobre o sinal do chifre de búfalo foi encontrado.

### Análise Estatística

A análise estatística utilizou a versão 18.0 do programa PASW Statistics para Windows. Valores de *p* menores do que 0,05 foram considerados significativos. Variáveis nominais foram analisadas pelo teste do Qui quadrado (χ ^2^ ) ou pelo teste exato de Fisher quando > 20% das células tinham contagem esperada menor do que 5.

## Resultados

O sinal do chifre de búfalo foi encontrado em 13 dos 28 casos (46,4%), sendo o terceiro sinal mais prevalente depois do fragmento no interior da incisura intercondilar ( *n* = 21; 75,0%) e da ausência do sinal da gravata borboleta ( *n* = 17; 60,7%). O sinal ocorreu em 7/19 meniscos mediais (36,8%) ( Fig. 2 ) e em 6/9 meniscos laterais (66,7%) ( Fig. 3 ). Na presença de achados sugestivos de deslocamento anterior da alça ( *n* = 9), o novo sinal foi visto em 7 casos (77,8%), sendo 3 meniscos mediais (33,3%) e 4 meniscos laterais (66,7%). Na presença de achados sugestivos de um fragmento com deslocamento posterior ( *n* = 4), o sinal de chifre de búfalo foi observado em 2 pacientes (50,0%), 1 menisco medial e 1 lateral. Nenhum dos achados supramencionados foi estatisticamente significativo.

Uma associação significativa entre o novo sinal e um fragmento no interior da incisura intercondilar ( *p* = 0,01) e um sinal de corno anterior duplo ( *p* = 0,01) foi observada ( Tabela 2 ). Não houve outros achados estatisticamente significativos. O chifre de búfalo foi observado independentemente de uma ruptura do LCA ( Tabela 3 ).

**Tabela 2:** Frequência do sinal do chifre do búfalo na presença de outros achados

Sinal do chifre do búfalo						
Sinal	Presente ( *n* = 13): *n* (%)	Ausente ( *n* = 15): *n* (%)	Total ( *n* = 28): *n* (%)	RC (IC)	Teste estatístico	*p*
Fragmento intercondilar	13 (100%)	8 (53%)	21 (75%)	**0,53** **(0,33–0,86)**	**EF**	**0,01**
Ausência do sinal de gravata borboleta	10 (77%)	7 (47%)	17 (60%)	3,81 (0,74–19,67)	χ ^2 ^ = 2,67	0,10
Sinal em V	6 (46%)	3 (20%)	9 (32%)	3,43 (0,65–18,22)	EF	0,23
LCP duplo	6 (46%)	2 (13%)	8 (28%)	5,57 (0,88–35,27)	EF	0,10
Menisco invertido	6 (46%)	2 (13%)	8 (29%)	5,57 (0,88–35,27)	EF	0,10
Corno anterior duplo	5 (39%)	0 (0%)	5 (18%)	**1,63** **(1,06–2,50)**	EF	**0,01**
Corno posterior desproporcional	5 (39%)	2 (13%)	7 (25%)	4,06 (0,63–26,13)	EF	0,20
LCA duplo	2 (15%)	0 (0%)	2 (7%)	1,18 (0,94–1,49)	EF	0,21
Sinais indiretos						
Efusão articular	10 (77%)	7 (47%)	17 (60%)	3,81 (0,74–19,66)	χ ^2 ^ = 2,67	0,10
Edema da medula subcondral	5 (39%)	4 (27%)	9 (32%)	1,72 (0,35–8,51)	EF	0,69
Edema linear da medula subcondral	3 (23%)	5 (33%)	8 (29%)	0,60 (0,11–3,21)	EF	0,69
Edema subcondral do PTM	3 (23%)	6 (40%)	9 (32%)	0,45 (0,09–2,35)	EF	0,44
Extrusão de menisco	2 (15%)	5 (33%)	7 (25%)	0,36 (0,06–2,31)	EF	0,40
Cisto parameniscal	0 (0%)	2 (13%)	2 (8%)	0,88 (0,71–1,06)	EF	0,48
Outras lesões						
Ruptura de LCA	5 (39%)	6 (40%)	11 (39%)	0,94 (0,21–4,29)	χ ^2 ^ = 0,01	0,93
Hemialça de balde	0 (0%)	3 (20%)	3 (11%)	0,80 (0,62–1,03)	EF	0,23

**Abreviaturas:** χ ^2^ , teste do Qui quadrado; EF, teste exato de Fisher; LCA, ligamento cruzado anterior; LCP, ligamento cruzado posterior; PTM, platô tibial medial; RC, razão de chances.

**Notas** : Foi observada uma associação entre o fragmento no interior da incisura intercondilar e o corno anterior duplo com o novo sinal relatado. Os testes χ ^2^ e EF foram realizados. Os valores de *p* estatisticamente significativos estão em negrito.

**Tabela 3:** Ruptura do ligamento cruzado anterior e frequência dos sinais da ruptura em alça de balde

Ruptura do LCA						
Sinal	Presente ( *n* = 11): *n* (%)	Ausente ( *n* = 17): *n* (%)	Total ( *n* = 28): *n* (%)	RC (IC)	Teste estatístico	*p*
Fragmento intercondilar	7 (64%)	14(82%)	21 (75%)	0,38 (0,07–2,16)	EF	0,38
Ausência do sinal de gravata borboleta	8 (73%)	9 (53%)	17 (60%)	2,37 (0,46–12,14)	EF	0,44
Chifre de búfalo	5 (46%)	8 (47%)	13 (46%)	0,94 (0,21–4,29)	χ ^2 ^ = 0,01	0,93
Sinal em V	4 (36%)	5 (29%)	9 (32%)	1,37 (0,27–6,87)	EF	1,00
LCP duplo	3 (27%)	5 (29%)	8 (28%)	0,90 (0,17–4,87)	EF	1,00
Menisco invertido	2 (18%)	6 (35%)	8 (29%)	0,41 (0,07–2,53)	EF	0,42
Corno posterior desproporcional	4 (36%)	3 (18%)	7 (25%)	2,67 (0,46–15,35)	EF	0,38
Corno anterior duplo	4 (36%)	1 (6%)	5 (18%)	9,14 (0,86–97,27)	EF	0,06
LCA duplo	0 (0%)	2 (12%)	2 (8%)	0,88 (0,74–1,05)	EF	0,51
Sinais indiretos						
Efusão articular	7 (64%)	10 (59%)	17 (60%)	1,22 (0,26–5,85)	EF	1,00
Edema da medula subcondral	6 (65%)	3 (18%)	9 (32%)	5,60 (1,01–31,32)	EF	0,38
Edema subcondral do PTM	6 (65%)	3 (18%)	9 (32%)	5,6 (1,00–31,32)	EF	0,10
Edema linear da medula subcondral	2 (18%)	6 (25%)	8 (29%)	0,41 (0,07–2,53)	EF	0,42
Extrusão de menisco	3 (27%)	4 (23%)	7 (25%)	1,22 (0,22–6,92)	EF	1,00
Cisto parameniscal	0 (0%)	2 (12%)	2 (8%)	0,8 (0,74–1,05)	EF	0,51
Outras lesões						
Hemialça de balde	0 (0%)	3 (18%)	3 (11%)	0,8 (0,66–1,03)	EF	0,26

**Abreviaturas:** χ ^2^ , teste do Qui quadrado;EF, teste exato de Fisher; LCA, ligamento cruzado anterior; LCP, ligamento cruzado posterior; PTM, platô tibial medial; RC, razão de chances.

**Notas:** Não houve diferença na prevalência dos achados entre os grupos com ou sem ruptura do LCA. Foram realizados os testes χ ^2^ e EF.

Observando os achados dos cortes axiais, o sinal em V foi identificado em 9 casos, o que indica uma sensibilidade de 32,1%. Destes nove pacientes, três apresentavam somente o sinal em V, e seis também apresentavam o sinal de chifre de búfalo. Dos 13 pacientes com este último, 7 tinham apenas o sinal do chifre de búfalo, mas não o sinal em V. Nenhum desses sinais foi o único sinal de ruptura, e todos os casos apresentavam simultaneamente o fragmento no interior da incisura intercondilar e o sinal de ausência da gravata borboleta.

A ruptura ocorreu no menisco medial em 19 casos (67,8%), e no menisco lateral, em 9 casos (32,2%). Três casos apresentam achados imagiológicos sugestivos de hemialça de balde, 27 mas tal não pôde ser confirmado pelos prontuários cirúrgicos, sendo que nenhum desses pacientes apresentou o sinal do chifre de búfalo. Seis exames de RM não diagnosticaram ruptura em alça de balde, mas todos apresentaram pelo menos um sinal indireto. O novo sinal nunca foi encontrado em um menisco não acometido.

## Discussão

**Fig. 4:**
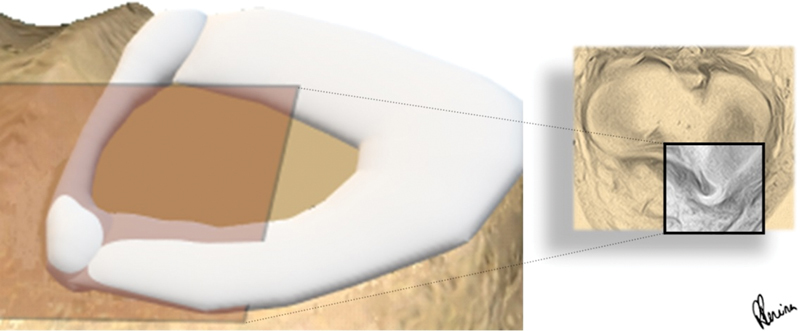
O fragmento deslocado em forma de alça de balde do menisco medial de um joelho direito em corte axial (caixa laranja), com aparência de chifre.

O achado mais relevante do presente estudo foi o de que o chifre de búfalo pode ser um novo sinal para o diagnóstico de rupturas em alça de balde do menisco. É útil para ambos os meniscos em fragmentos deslocados para anterior 18 20 ou posterior. 19 O sinal foi observado em 7/19 meniscos mediais (36,8%) e em 6/9 meniscos laterais (66,7%), sem diferença significativa. Todos os casos com duplo corno anterior tinham sinal de chifre de búfalo ( *p* = 0,01). Em 8/28 casos (28,6%) com menisco invertido, o sinal proposto apareceu em 6 (75,0%). Ambos os pacientes com duplo LCA também apresentam o sinal do chifre de búfalo (100%). O achado também apareceu em 2/4 casos com corno posterior desproporcional. Além disso, todos os pacientes com o novo achado tinham um fragmento dentro da incisura intercondilar ( *p* = 0,01). O novo sinal nunca apareceu sozinho, mas sempre na presença de outros sinais de deslocamento do fragmento. Assim, pode-se concluir que o sinal de chifre de búfalo indica uma alça deslocada de uma ruptura de menisco e que aparece quando o corte axial o bissecta ( Fig. 4 ).

**Fig. 5:**
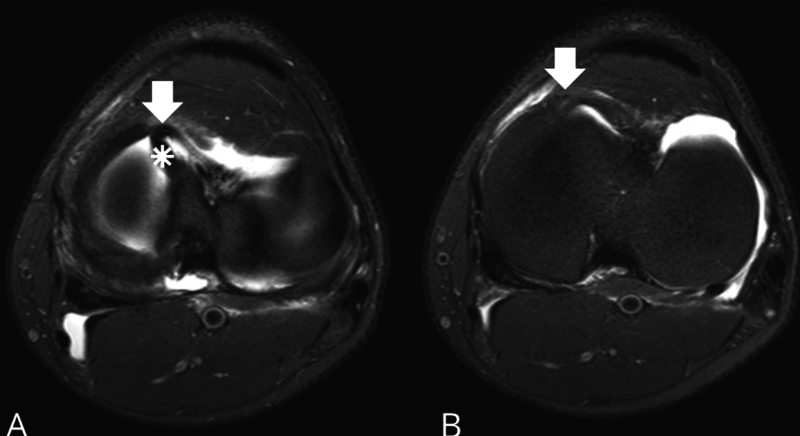
Cortes transversais de uma ressonância magnética pré-operatória de um homem de 27 anos com ruptura em alça de balde do menisco medial do joelho esquerdo. ( **A** ) O sinal em V (*) e o sinal do chifre de búfalo (seta) são visíveis. ( **B** ) No corte seguinte, apenas o sinal do chifre de búfalo (seta) é observado. Sequência T2 SPAIR axial: matriz, 300 × 250; TR/TE, 3.800/65 milissegundos; corte, 3 mm; *gap* , 0,3; média, 2; FOV, 180 mm; FA, 90°.

Este novo achado foi observado porque a avaliação de imagens em corte transversal está ganhando importância na prática clínica, e havia apenas um sinal previamente descrito para esta lesão nestas imagens: o sinal em V. 23 Apesar de próximos, esses dois achados não são os mesmos. O chifre de búfalo usa uma sequência de imagens, é anterior, e não precisa ter a alça e o menisco no mesmo corte. Sete pacientes tinham o sinal do chifre de búfalo, mas não o sinal em V, e três tinham o último, mas não o primeiro. Seis de nove pacientes apresentaram os dois sinais ao mesmo tempo ( Fig. 5 ). Nesta amostra, o sinal em V teve 32,1% de sensibilidade, valor inferior ao relatado por Rao et al., 23 de 72,0%, e menor do que a sensibilidade do sinal do chifre de búfalo nesta amostra (46,4%).

A sensibilidade geral da RM neste estudo foi baixa, e correspondeu à metade inferior do intervalo de sensibilidade relatado anteriormente, de 64% a 93%. 4 5 12 13 21 22 Ao todo, 6 de 28 exames de RM não detectaram qualquer sinal de ruptura em alça de balde do menisco, o que se traduz em uma sensibilidade geral de apenas 78,6%. Esse fato pode explicar a baixa sensibilidade do sinal do chifre de búfalo (46,4%), embora tenha sido o terceiro sinal mais relevante, com sensibilidade maior do que os outros 6 achados amplamente difundidos. Sua sensibilidade foi superada pelo fragmento no interior da incisura intercondilar (21/28; 75,0%) e pela ausência do sinal da gravata borboleta (17/28; 60,7%). Isso é consistente com a sensibilidade relatada por Dorsay e Helms para o fragmento dentro da incisura intercondilar de 76,7% 22 mas é menor do que os 88,4% descritos para o sinal da ausência da gravata borboleta no mesmo estudo. Nesta pesquisa, não se pretende calcular a especificidade ou a reprodutibilidade do sinal do chifre de búfalo, mas nunca o observamos em meniscos não acometidos; ademais, a visualização do sinal requer a presença de um fragmento deslocado. Portanto, acreditamos em sua especificidade. Além disso, a definição proposta e a aparência do sinal do chifre de búfalo são fáceis de detectar, talvez mais do que o fragmento no interior da incisura intercondilar ou do que a ausência da gravata borboleta, cujas definições são vagas.

Este estudo tem várias limitações. A amostra foi pequena devido aos obstáculos, como a informatização limitada de arquivos médicos anteriores a 2012 e o baixo volume de pacientes tratados. Embora isso tenha impedido a obtenção de uma amostra maior, acreditamos que não ameaça o objetivo deste artigo. Os meniscos não acometidos foram usados como controles, e o tempo decorrido entre o trauma, a RM e a cirurgia pode ter sido uma fonte de viés. Os pacientes foram escolhidos a partir de um diagnóstico cirúrgico, o que não permitiu o teste de especificidade. A confiabilidade do sinal não foi calculada. A amostra reúne RM com diferentes intensidades de campo magnético, mas Van Dyck et al. 30 mostraram, em um estudo prospectivo controlado, que a precisão diagnóstica de 3,0 T para rupturas do menisco e do LCA não é significativamente maior em comparação a 1,5 T. Assim, parece razoável acreditar que essa questão não compromete a identificação de um novo sinal. Joelhos com deficiência de LCA (aguda ou crônica; 11/28; 39,3%) e joelhos com LCA competente foram estudados, mas há controvérsias quanto ao seu efeito na precisão da RM. 10 12 Embora todas as cirurgias tenham sido realizadas por cirurgiões de joelho experientes que trabalham no mesmo departamento, os relatos cirúrgicos não eram padronizados, e alguns não incluíam a classificação do subtipo de ruptura em alça de balde.

## Conclusão

Em conclusão, o sinal do chifre de búfalo pode estar presente em rupturas de meniscos mediais e laterais acompanhadas ou não por rupturas do LCA. O sinal é provocado por um fragmento deslocado e, assim, está estatisticamente associado ao fragmento no interior da incisura intercondilar e ao sinal do chifre duplo anterior. Reconhecer essa situação é muito importante para determinar o tipo de tratamento e planejar a cirurgia. Além disso, acreditamos que esse achado é fácil de identificar e é específico para rupturas em alça de balde, por não ter sido encontrado em meniscos não acometidos.

Aqui, o sinal do chifre de búfalo aparece como um novo achado que pode ser relevante para o diagnóstico de rupturas em alça de balde do menisco usando imagens de RM em cortes axiais.
